# Teachers’ role in digitalizing education: an umbrella review

**DOI:** 10.1007/s11423-022-10166-0

**Published:** 2022-10-31

**Authors:** Olivia Wohlfart, Ingo Wagner

**Affiliations:** Institute for School Pedagogy and Didactics, Karlsruhe Institute of Technology (KIT), Kaiserstraße 12, 76131 Karlsruhe, Germany

**Keywords:** TPACK, Digital literacy, Technology integration, Strategies

## Abstract

As teachers are central to digitalizing education, we summarize 40 years of research on their role in that process within a systematic umbrella review that includes 23 systematic reviews with a total of 1062 primary studies focusing technology integration and aspects of digital literacy. Our findings highlight the international acceptance of the TPACK framework as well as the need for a clear concept of digital literacy. It is unique that we identify and discuss parallels in developing teachers’ digital literacy and integrating digital technologies in the teaching profession as well as barriers to those goals. We conclude by suggesting future directions for research and describing the implications for schools, teacher education, and institutions providing professional development to in-service teachers.Kindly check and confirm whether the corresponding author is correctly identified.Olivia Wohlfart is correctly identified as corresponding author.

## Introduction

A variety of stakeholders must be mutually committed to creating digitally competent schools (Pettersson, 2018; Sailer et al., 2021), and teachers are seen as crucial to this process of digitalization (Bridwell-Mitchell, 2015; Lockton & Fargason, 2019). Moreover, the role of teachers in digitalizing education must be recognized as a complex, holistic phenomenon (Ertmer & Ottenbreit-Leftwich, 2010). Teachers can be a driving force of digitalization, but the COVID-19 pandemic and associated distance teaching/learning have also made teachers prisoners of the rapid digitalization of society and of the associated expectations for education as they are forced to use digital technologies (Wohlfart et al., 2021). Before 2020, some institutions were still discussing data protection guidelines while others were already trying to “crack the code of education reform” (Tienken & Starr, 2020). By 2021, this situation had changed entirely, and distance learning and digitalization became inescapable, yet only 41% of teachers internationally reported having learned how to integrate digital technologies into teaching (Drossel et al., 2019; IEA, 2019). While policy and organizational infrastructure are pivotal in successfully promoting the digitalization of education, research has shown that teachers’ digital literacy is more important in that process than rich access to digital technologies (Pettersson, 2018).

Previous research on the role of teachers in this process has often focused either on their (perceived) digital literacy or on their willingness and ability to integrate technology (e.g., Granić & Marangunić, 2019; McKnight et al., 2016). Various models have been developed to examine the digital literacy of teachers and teacher educators, the most prominent being the Technological-Pedagogical-Content-Knowledge (TPACK) model (Koehler & Mishra, 2008; Mishra & Koehler, 2006), which acknowledges the complexity of teaching by differentiating seven knowledge domains in the interplay of technological, pedagogical, and content knowledge. Since the model’s first publication in the mid-2000s, the international scientific community has directed much attention and encouragement but also criticism toward it. To date, the original article by Mishra and Koehler (2006) has been cited over 10,000 times (Google Scholar).

Due to global trends of digitalization, the literature on digitalization in education has flourished in recent decades, occasioning a number of literature reviews in this crowded field. As the number of publications per year relentlessly increases, it has become difficult to stay abreast of current findings, but literature reviews have the advantage of systematically structuring and summarizing the previous literature on a specific topic (Mullins et al., 2014). Because teachers are central to implementing digitalization, this second-order review study aims to examine the (main) research focus of previous reviews related to teachers’ perspectives on the digitalization of school education and to identify future directions for research on the role of teachers in this process. Due to varying theoretical approaches and research questions, timeframes and sample groups, previous reviews on teachers’ role on the digital transformation often focus very specific aspects of these. It is unique to this approach, that we are able to identify parallels and connections between overarching themes which have been examined independently in the past. With this holistic overview of research on the digitalization of education from a teachers’ perspective, we aim to answer the following research questions:

RQ1: What is the (main) research focus of previous reviews concerning teachers’ role in the digitalization of school education?
RQ2: What is the current state of research on the digital literacy of teachers?
RQ3: What is the current state of research on the role of teachers in technology integration?
RQ4: What are the future directions for research focusing on the role of teachers in the digitalization of school education?

To answer these research questions, literature reviews and meta-analyses with a focus on teachers and digitalization were examined by means of a systematic umbrella review.

## Method

An abundance of research on teachers and the digitization of education has been conducted in the past decades. Systematic reviews and meta-analyses offer context-specific overviews and critical reviews of these studies and add to our knowledge base. Our goal is to refine this knowledge base by combining these reviews “under one umbrella.” Instead of repeating searches, assessing the study eligibility of included articles, etc., we provide a systematic overview and critical review of research on a complex topic, following the protocol recommended for umbrella reviews by the Joanna Briggs Institute (Aromataris et al., 2015). Furthermore, we analyze whether, and discuss how, independently derived conclusions and discussions of these reviews align.

### Inclusion criteria

In our umbrella review, we refer to syntheses of research evidence, including systematic reviews and meta-analyses focusing on pre- and in-service teachers’ digital literacy as well as their application of technology-based education in primary and secondary education. Due to the emerging nature of our research topic, we include all available review types and articles (Grant & Booth, 2009).

### Search procedure

The search was conducted using the search engine EBSCOhost and included the databases Education Resource Complete, Academic Search Complete, and Education Resources Information Center. To ensure the quality of the syntheses, only articles and reviews published in peer-reviewed journals were included. For better reproducibility, we opted for articles in English language as the *lingua franca* in the global, scientific community. The selected search terms were determined by means of an exploratory literature analysis of scientific and educational policy documents as well as the authors’ expertise.

**Fig. 1:**
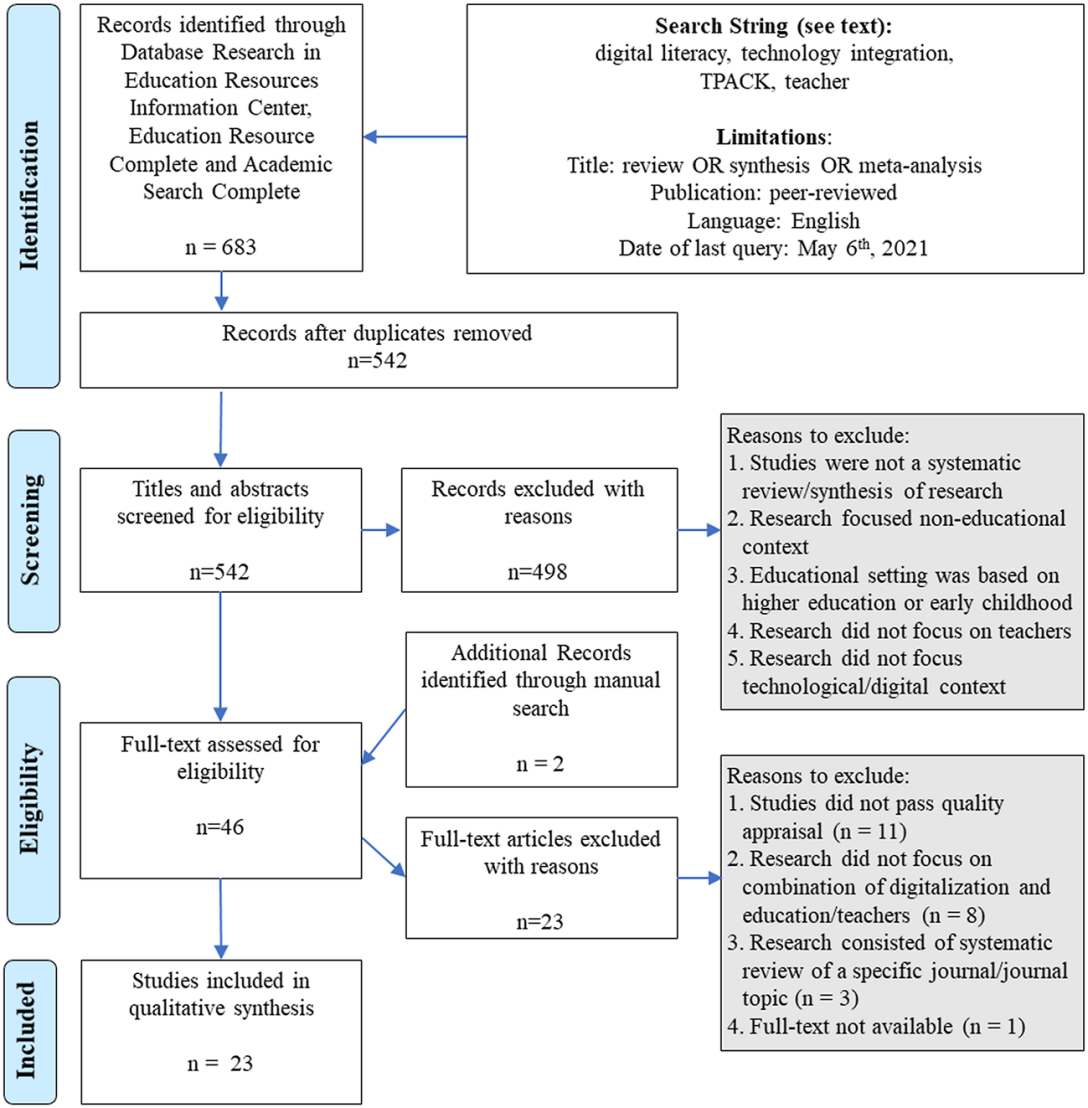
Flow diagram of the literature search and selection of eligible reviews (adapted from the PRISMA Statement; Moher et al., 2009)

In a first search attempt, we used various synonyms of the terms “digital literacy” and “digital competence” as well as “technology integration” and “educational technology,” with the addition of “teachers” and various “review” methods. As this yielded over 20,000 results, we refined the search string to focus on teachers’ digital literacy and integration of technology. This resulted in the following Boolean search phrase: (“digital literac*” OR “digital competenc*” OR “ICT skill*” OR “digital skill*” OR “computer skill*” OR “technological skill*” OR “e-literac*” OR “multi-modal skill*” OR (“technology” AND (“implementation” OR “integration” OR “application”)) AND teacher* AND (review OR synthesis OR meta-analysis). A total of 9,080 results were identified in the search (date of last search: May 6, 2021). To further reduce the number of articles to a manageable amount, we adapted our search string to consider only studies including “review,” “synthesis,” or “meta-analysis” in the title, which yielded a total of 683 results across the three databases. After duplicates were removed, 542 studies were submitted for further title and abstract screening. Figure 1 summarizes the search (identification) and eligibility steps (screening and checking).

### Study selection

All the identified articles were examined by two researchers through an initial screening of titles and abstracts based on the inclusion and exclusion criteria. This resulted in the exclusion of 498 publications. We excluded articles that did not conduct a systematic review or meta-study as well as those lacking an educational, digital, or teacher-centered focus. Articles focusing on studies of early childhood or higher education were also excluded from further analysis.

Of the selected 44 articles, we were not able to access one paper and received no positive response after reaching out to the authors via email. Furthermore, we conducted hand searches of pertinent academic journals in the field and of the reference lists of the identified articles and extracted two additional papers: Rokenes and Krumsvik (2014) and Wang et al. (2018). In summary, 45 articles were read in full text and assessed for eligibility based on the a priori inclusion and exclusion criteria:

1. *Context* the study examined digitization in the context of teaching and learning.
2. *Teacher sample* the study targeted pre- or in-service teachers in primary or secondary education.
3. *Methodological quality* the study was a systematic review or meta-study.

The decision to exclude full-text articles was made by the first author in discussion with the second author. Upon reading the full texts, 11 articles were excluded due to the context or sample of the study.

Next, the methodological quality of the remaining 34 articles was assessed with an appraisal checklist based on the JBI Critical Appraisal Checklist for Systematic Reviews and Research Syntheses (Aromataris et al., 2015; Moher et al., 2009) as well as Gessler and Siemer (2020). Only articles that at least partially met all the appraisal criteria were included in the subsequent qualitative synthesis of our umbrella review. Eleven articles did not meet the minimum requirements and were excluded from further analysis.

In total, we included 23 articles in our qualitative synthesis based on extensive screening and assessment of the identified records (Fig. 1). Except for two meta-analyses, the conducted studies are categorized as systematic reviews with narrative overviews of the state of research on the given topic.

### Data analysis

To answer the research questions, we conducted a quantitative and qualitative content analysis of the 23 systematic reviews. For the quantitative analysis, a protocol was developed for categorizing the general characteristics (publication site, research design, included studies, research objective(s)/questions). This was followed by a content-based thematic analysis of the 23 articles to identify latent patterns, themes, and subthemes through an iterative reading and coding process (Braun & Clarke, 2006) supported by MAXQDA software. The identified themes were discussed by the team of authors and then recoded by the first author. Finally, 16 categories (with varying numbers of subcategories) were identified from 1780 coded posts.

## Quantitative results

The umbrella review included 23 research articles from 18 scientific journals, published between 2006 and 2020. Without regard to possible duplicates, we found 1321 studies within the reviews.^1^ We identified the overlapping studies among the reviews and determined that this umbrella review includes 1062 studies.

**Fig. 2:**
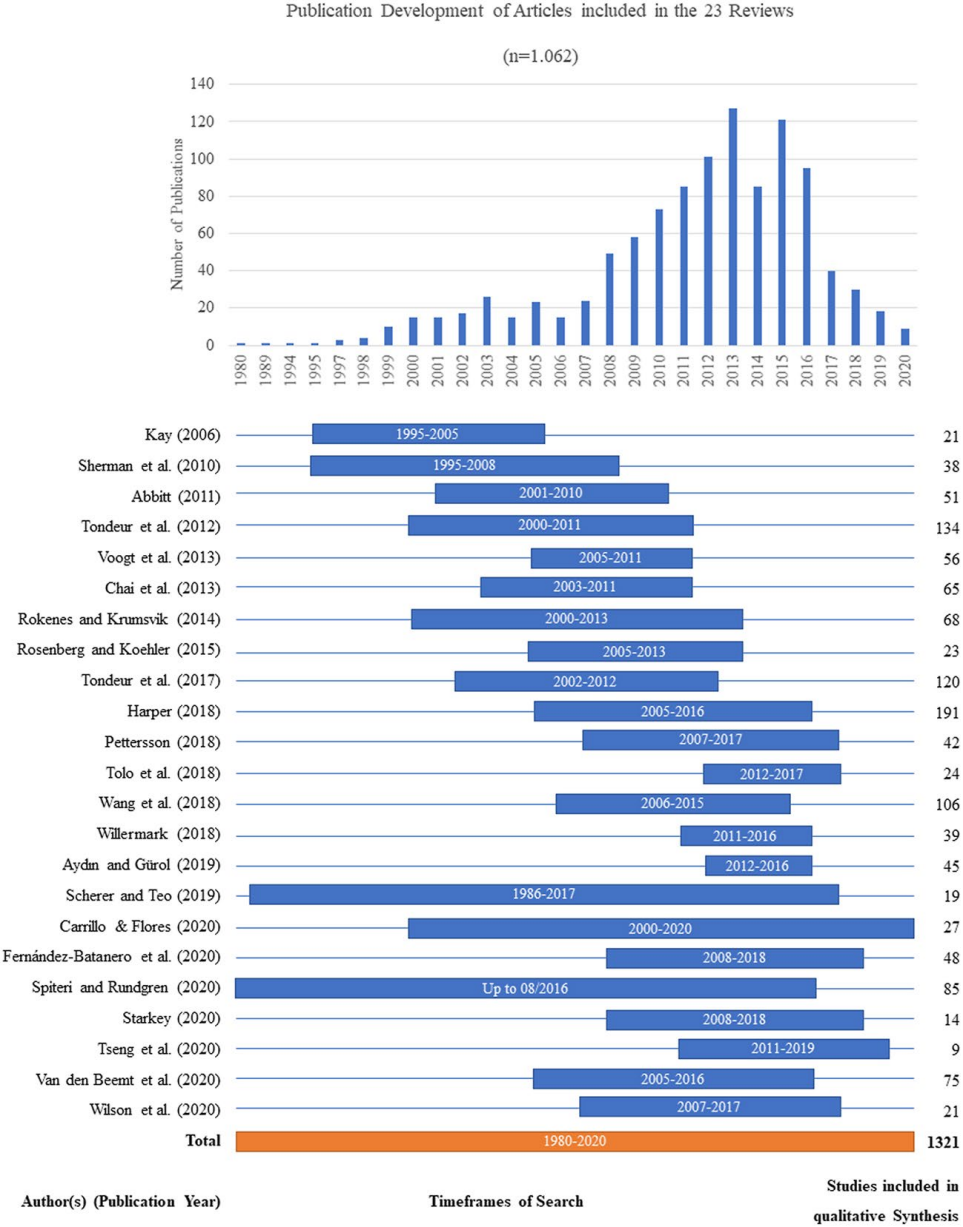
Publication development of articles included in the selected reviews (n = 23)

The reviews included studies published between 1980 and 2020 (Fig. 2). We found that several authors were mentioned and included repeatedly: Chai, Koh, Koehler, Mishra, Polly, and Tondeur. We also found overlap for several publications; e.g., the study by Niess (2005) was included in seven of the reviews, six studies were included in five reviews, and a further 14 studies appeared in four reviews. Notwithstanding, 84% of the studies (890) were included in only one review.

## Qualitative findings

The qualitative analysis was guided by the formulated research questions. In "Research focus of previous reviews" section, we provide an overview of the main research foci of the included reviews (RQ 1). Next, we describe the current state of research on teachers’ digital literacy ("Digital literacy" section RQ 2) and their (supposed) role in the integration of technology ("Technology integration"section RQ 3). Finally, in "Future research" section, we identify relevant areas for future research, focusing on the role of teachers and their digital literacy in the digitalization of school education (RQ 4).

### Research focus of previous reviews

In regard to RQ 1, we identified six themes as main research foci of previous reviews on the digitalization of school education from the perspective of teachers:

1. Digital Literacy,
2. Teacher Preparation (Programs),
3. Role of Teachers,
4. Institutional Environment,
5. Technology Integration, and.
6. Technology as Tools.

The most prominent theme, which was included in over half of the reviews, concerned teachers’ digital literacy (n = 14). Within these reviews, methods and instruments which assessed and discussed digital literacy of teachers were analyzed (e.g. Rosenberg and Koehler (2015) critically reflect how context is considered in TPACK research). The role and responsibilities of teacher preparation (programs) was addressed in eleven of the reviews, often in combination with a demand for a better preparation concerning digital literacy (e.g. Rokenes & Krumsvik, 2014). Several reviews also focused the critical role of teachers (n = 11) and/or the institutional environment (n = 9) in the process of digital transformation within the education system, highlighting the need for a holistic analysis on digitalization of school education and reliance on further stakeholders (e.g. Pettersson, 2018). Critical factors and requirements for successful technology integration were included and discussed in seven of the reviews. Finally, we identified a sixth theme which examined (specific) technologies as tools which influence and support student learning as well as interaction between teachers and students (Harper, 2018). Table 1 offers an overview of the main research focus of all 23 reviews as well as the identified themes included within these.

**Table 1:** Overview and research focus of selected reviews on teachers’ role in the digitalization of education (n = 23)

Author(s) and year of publication	Number of studies included	Study designs included	Sample	Main research focus	Themes included						Theoretical framework			
					Digital literacy	Teacher preparation (Programs)	Role of teachers	Institutional environment	Technology integration	Technology as tools	TPACK		Other^a^	None
Abbitt (2011)	20	n.a.	Preservice teachers	Examines the emerging methods and instruments designed to assess the TPACK of preservice teachers.	x	x					x			
Aydın and Gürol (2019)	65	Quantitative (n = 45), qualitative (n = 9), mixed methods (n = 11)	Teachers, students, administrators, and schools	Summarizes critical factors pertinent to ICT use in education and discusses directions for future ICT research.	x				x					x
Carrillo and Flores (2020)	134	Qualitative (n = 53), quantitative (n = 30), mixed methods (n = 51)	Preservice and in-service teachers, teacher educators, and other academic staff	Analyzes how and why online teaching and learning occurs in teacher education and explores its implications.			x			x			x	
Chai et al. (2013)	74	Qualitative (n = 31), quantitative (n = 13), mixed methods (n = 11), non–data-driven (n = 19)	TPACK research	Consolidates research concerning emerging trends, findings, and issues generated in TPACK studies and identifies future research needs.	x		x				x			
Fernández-Batanero et al. (2020)	21	Qualitative (n = 8), quantitative (n = 6), mixed methods (n = 7)	Primary, high school, and university teachers	Identifies research trends and directions for future research on digital competencies and teacher professional development.	x	x		x						x
Harper (2018)	25	Qualitative (n = 16), quantitative (n = 4), mixed methods (n = 3), theoretical (n = 2)	K-12 educational settings	Synthesizes previous research on how technology influences interactions between teachers and students.			x			x				x
Kay (2006)	68	Surveys (n = 30), qualitative (n = 11), mixed methods (n = 8), n.a. (n = 19)	Preservice teachers	Identify, illustrate and evaluate strategies used to incorporate technology into preservice education.	x	x		x	x					x
Pettersson (2018)	41	n.a.	Diverse educational contexts	Reviews research on pedagogical aspects of digital competence “in terms of policy, organizational infrastructures, strategic leadership as well as teachers and their teaching practices” (p. 1007).	x	x	x	x						x
Rokenes and Krumsvik (2014)	42	Qualitative (n = 24), quantitative (n = 7), mixed methods (n = 11)	Preservice teachers (secondary education)	Reviews knowledge about empirical research on ICT training in teacher education and discusses implications for teacher education institutions.	x	x								x
Rosenberg and Koehler (2015)	70	n.a.	TPACK research	Synthesizes whether and how context is considered in current TPACK research.	x			x			x			
Scherer and Teo (2019)	46	Quantitative (k = 51 correlation matrices)	In-service and preservice teachers	Synthesizes and discusses quantitative research evidence on the structural relations among core variables of the TAM for teachers.					x				x	
Sherman et al. (2010)	24	n.a.	Middle school education/teaching	Identifies and describes teaching methods and content that characterize middle school technology education teaching practice.			x	x						x
Spiteri and Chang Rundgren (2020)	27	Quantitative (n = 11), qualitative (n = 10), mixed methods (n = 6)	Primary education	Synthesizes previous research on the use of digital technology to illustrate factors affecting technology integration.	x		x		x	x				x
Starkey (2020)	48	Qualitative (n = 24), quantitative (n = 7), mixed methods (n = 11)	Preservice teachers	Synthesizes previous studies examining the preparation of teachers for the digital age and identifies future research.	x	x		x			x^b^			
Tolo et al. (2018)	10	n.a.	Teachers	Summarizes how technology does or does not assist teachers in their formative assessment practice.			x			x			x	
Tondeur et al. (2017)	14	Qualitative	Teachers	Synthesizes previous qualitative research on the relationship between teachers’ pedagogical beliefs and their uses of technology.			x		x					x
Tondeur et al. (2012)	19	Qualitative	Preservice teachers	Presents and discusses strategies for contemporary technology integration in preservice teacher education programs based on qualitative research.		x		x	x					x
Tseng et al. (2020)	51	Qualitative, quantitative, and mixed methods	In-service and preservice language teachers, students, and teacher educators	Identify and discuss TPACK research focusing on language teachers.	x	x					x			
van den Beemt et al. (2020)	271	Quantitative (n = 112), qualitative (n = 65), mixed methods (n = 84), other (n = 10)	n.a.	“Enable teachers to make a well-considered and evidence-based choice for the pedagogical use of social media in their classrooms, while taking into account … three levels of the curriculum” (p. 3).			x	x		x				x
Voogt et al. (2013)	55	Empirical (n = 44), theoretical (n = 11)	TPACK research	”Investigate the theoretical basis and the practical use of TPACK” (p. 2).	x		x	x			x			
Wang et al. (2018)	85	Qualitative, quantitative, and mixed methods	Preservice teachers	Synthesizes the literature describing preservice teachers’ development of TPACK.	x	x					x			
Willermark (2018)	107	Quantitative (n = 50), mixed methods (n = 44), qualitative (n = 13)	In-service and preservice teachers	Reviews the characteristics of empirical TPACK studies based on TPACK as knowledge vs. competence.	x	x	x				x			
Wilson et al. (2020)	38	Quantitative studies (k = 46 independent effect sizes)	Preservice teachers	Examines the effects of teacher education courses for technology integration on (practical and conceptual) teacher knowledge.		x			x		x			
*n.a.* not applicable														
^a^Carrillo and Flores (2020) apply the Community of Inquiry Framework (Garrison et al., 1999) as an analytical tool, Scherer and Teo (2019) analyze and discuss the variables of the TAM (Davis, 1986) through a meta-analysis, and Tolo et al., (2018) consider aspects of classroom assessment practices under their theoretical framework, “Assessment for Learning” (Hopfenbeck et al., 2015).														
^b^Starkey (2020) repeatedly refers to the TPACK model without applying the framework as a specific theoretical background.														

To better understand and classify the diverse foci of the reviews, we examined the theoretical frameworks as applied or recognized by the author(s). In 11 reviews, no specific theoretical framework was applied (cf. Table 1). Eight reviews based their work specifically on the TPACK framework. Three further frameworks were applied in individual studies; Carrillo and Flores (2020) used the Community of Inquiry Framework (Garrison et al., 1999) as an analytical tool, Scherer and Teo (2019) analyzed and discussed the variables of the technology acceptance model (TAM) (Davis, 1986) in their meta-analysis, and Tolo et al. (2018) considered aspects of classroom assessment practices under their own theoretical framework, “Assessment for Learning” (Hopfenbeck et al., 2015).

### Digital literacy

To answer RQ2, we analyzed how teachers’ digital literacy was approached in the reviews and considered their main findings. This topic was a thematic focus of 14 of the 23 systematic reviews, including 10 reviews that applied the TPACK framework. We present the findings of our qualitative analysis related to the individual and the assumed concept of digital literacy (4.2.1), TPACK (4.2.2), approaches to developing teachers’ digital literacy (4.2.3), and prevalent requirements (4.2.4).

#### Concept of digital literacy

The reviews offer a variety of definitions of digital literacy from policy papers and scientific studies alike. Rokenes and Krumsvik (2014, p. 252) follow a definition of digital literacy from Scandinavian studies on ICT in education and include “skills, knowledge, creativity and attitudes” in respect to digital media. Spiteri and Chang Rundgren (2020) include areas of digital literacy as proposed by the European Commission’s framework for developing and understanding digital competence in Europe (Ferrari, 2013; Starkey, 2020) differentiates three types of digital competency for teachers: generic digital competency, digital teaching competency, and professional digital competency. The reviews focusing on TPACK, meanwhile, present the original concept of the framework as introduced by Mishra and Koehler (2006).

#### TPACK

Eight reviews specifically focus on the TPACK framework and examine various aspects of previous research, including publication development, the distinction between TPACK knowledge domains, the measurement of TPACK, the interplay between context and TPACK, and model development and TPACK development (Table 2).

**Table 2:** Thematic overview of research on TPACK (n = 8)

Authors (Year of publication)	Publication development	Distinction of knowledge domains	Measuring TPACK	TPACK and context	Model development	Developing TPACK
Abbitt (2011**)**	x		x	x		
Chai et al. (2013)	x	x	x	x	x	x
Rosenberg and Koehler (2015**)**	x			x		
Starkey (2020**)**		x		x		
Voogt et al. (2013)	x	x	x	x		x
Wang et al. (2018)	x	x	x	x		x
Willermark (2018**)**	x		x	x	x	
Wilson et al. (2020)		x	x	x		x

The reviews report (in broad agreement) on the emergence and publication development of the TPACK model based on the original contribution of Shulman (1986) and the contributions of Mishra and Koehler (Koehler & Mishra, 2008; Mishra & Koehler, 2006). In addition, the studies of Pierson (2001) and Niess (2005) play a special role. These emerged shortly before and concurrently with the TPACK model, respectively, and refer to TPCK as “technology-enhanced” PCK.

Concerning the distinction of knowledge domains, four reviews specifically acknowledge that a clear definition and delineation of individual knowledge domains is rare and nearly impossible. They also concur that clear definitions and operationalization of knowledge domains would be helpful in (further) developing both the theoretical model and individual survey instruments. The reviews often report TPACK as an overarching knowledge domain. Nevertheless, individual reviews refer to specific knowledge domains, with technical knowledge (TK) taking a special role, as it strongly correlates with the development of TPACK (Wang et al., 2018). TK was defined in various ways and aligned with specific technologies (both analog and digital) or types of knowledge (Voogt et al., 2013), which points to challenges in distinguishing domain-specific from domain-unspecific technologies (Chai et al., 2013) as well as their dynamic and changeable nature over time (Abbitt, 2011; Voogt et al., 2013; Wang et al., 2018).

The most prominent topic discussed in the TPACK reviews is how to measure teachers’ TPACK. Five of the reviews present approaches and instruments for identifying and measuring TPACK, distinguishing between self-assessment and performance assessment, the former being applied in the large majority of studies. The survey instrument developed and validated by Schmidt et al. (2009) to measure self-perceived TPACK is explicitly highlighted in five of the eight reviews. In addition to quantified surveys, these studies also mention interviews, open-ended questions (mostly in the context of student teaching), interventions (with pre/post survey designs), reflective questionnaires, and document analyses as possible data collection methods. In addition to self-assessment, the reviews acknowledge that performance assessment by experts or peers plays an important role in measuring TPACK; such assessment applies either quantitative or qualitative content analysis (or both) to evaluate observations, reflection sheets, interviews, and classroom materials.

Overall, although they agree on the importance of context in connection with TPACK, the reviews treat this topic rather marginally as a limitation or area for further research and thus refer predominantly to school types, subject areas, pedagogical approaches, and the characteristics and beliefs of teachers. An exception is Rosenberg and Koehler’ (2015) context-specific review, which discusses the meaning and presence of context in TPACK research based on Porras-Hernández and Salinas-Amescua’s (2013) conceptual framework for context at three levels (micro, meso, and macro) and among two groups of actors (teachers and students). The authors conclude that context is often missing from research on TPACK and, when included, differs greatly in definition. Additionally, Chai et al. (2013) propose the “Technological Learning Content Knowledge” (TLCK) framework as a revision of the TPACK framework to include the learner perspective, addressing criticism of the examined studies and contributing to the further development of the model. Analogously, Willermark (2018) introduces the category of “TPACK as knowledge” versus “TPACK as competence” and examines the extent to which prior studies interpreted TPACK. Based on the results of her review (finding that most previous studies adopted the former perspective), she recommends adopting a changed perspective that understands and examines TPACK as a competence that can be developed and transferred (Willermark, 2018).

#### Approaches to developing teachers’ digital literacy

Ten of the reviews highlight best-practice examples of developing teachers’ digital literacy/TPACK within teacher preparation programs and professional development programs. The most promising approach to developing digital literacy appears to be (role) modelling (in 7 reviews). Rokenes and Krumsvik (2014) describe this approach as involving “teacher educators, in-service teachers, mentors, and peers promoting particular practices and views of learning through intentionally displaying certain teaching behavior, which could play an important role in shaping student teachers’ professional learning” (p. 262). A significant advantage for preservice teachers is the transferability of this approach to authentic classroom situations (Kay, 2006). The role of teacher educators and their training is also highlighted in this context (Tondeur et al., 2012), as poor modelling on the part of teacher educators may negatively impact preservice teachers’ TPACK development (Wang et al., 2018).

In addition to modelling, collaboration is considered to be important in developing teachers’ digital literacy and enhancing it in various formats; this was examined among preservice teachers, preservice teachers and teacher educators, in-service teachers, and in-service teachers and their students. In this context, the social dimensions of knowledge creation are repeatedly highlighted as important elements in increasing digital literacy.

Authentic learning situations are also highlighted as fruitful elements in developing teachers’ digital literacy (in 5 reviews). In discussing TPACK, Willermark (2018) argues that the authenticity of learning situations is decisive in the development of (theoretical) knowledge vs. (practical) competence and strongly recommends applying authentic approaches in learning situations to empower teachers both to be digitally literate and to have the skills to apply specific tools in their teaching.

Further strategies to develop teachers’ digital literacy include metacognition as reflection on action, bridging the theory/practice gap, learning by doing, implementing diverse assessment strategies, and blended learning. While the reviews present a variety of strategies, the success or effectiveness of these measures in developing teachers’ digital literacy is seldom reported.

#### Requirements for developing teachers’ digital literacy

Several reviews critically reflect on the requirements for developing teachers’ digital literacy, highlighting the importance of teacher preparation, the institutional environment, and the role of teachers. The reviews strongly agree on the need to integrate approaches to develope digital literacy in both teacher education (n = 6) and teacher professional development (n = 6) to prepare teachers for digitalized schools. In light of this, digitally literate teacher educators are indispensable in teacher preparation. Tondeur et al. (2012) recommend the development and maintenance of a technology plan for teacher education that considers both technical and instructional circumstances, with the ultimate goal of empowering end users.

Furthermore, the reviews report that institutional environment significantly affects success in developing digital literacy in various arenas, including leadership (n = 5), the policy debate (n = 4), and school culture (n = 2). Pettersson (2018) concludes that school leaders are pivotal in translating policies on digital literacy into specific goals and support actions at schools and contends that a failure to do so is the “main barrier for transforming ICT-policies into system-wide professional development and educational change” (p. 1013). A supportive policy debate at the local and national level is also reported as a requirement for enabling the development of preservice teachers’ digital literacy in the context of their teacher preparation (Wilson et al., 2020) as well as that of in-service teachers in the context of teacher professional development (Sherman et al., 2010). Analogously, a supportive school culture is described as a requirement, especially in further developing in-service teachers’ digital literacy (Spiteri & Chang Rundgren, 2020).

A final identified factor in developing digital literacy is the teachers’ role in the process. In the reviews, we identified four areas that directly impact digital literacy and its development: pedagogical beliefs (n = 11), personal characteristics (n = 7), interaction with students (n = 6), and experience with technology (n = 3). While not all these items can be directly influenced, the results highlight two main findings: (1) the evidence shows no differences in developing digital literacy between in-service and pre-service teachers (dispelling the myth of digital natives); (2) introducing and promoting a student-centered, constructivist pedagogical approach in teacher education positively influences the development of digital literacy.

### Technology integration

To answer the third research question, we examined whether and how the reviews discussed the integration and application of technology from the teachers’ perspective. We identified seven reviews which focus aspects of technology integration. The qualitative analysis highlights the relevance of specific strategies, requirements, and barriers to technology integration (4.3.1) as well as various facets of technology acceptance (4.3.2).

#### Strategies, requirements, and barriers to technology integration

The strategies and requirements for technology integration often mirror approaches to developing digital literacy. According to the qualitative findings, technology integration is influenced by the availability of technical support and facilitation, access to resources, paths to professional development, accurate pedagogical approaches, teachers’ digital literacy, possibilities of collaboration, leadership, and teacher educators. The review authors consent that integrating technology for the first time or integrating new technology requires knowledge of and access to these tools and, furthermore, time to explore them. Wilson et al. (2020) examine *knowledge* as key to a better integration of technology and highlight the relevance of specific teacher education courses for technology integration. In this sense, Spiteri and Chang Rundgren (2020) also underline the time allocated to training and teachers’ perceived support from school as two of the most influential factors in integrating technology. After access and time constraints, teachers’ attitudes or personal fears are repeatedly depicted as negatively affecting technology integration. Additionally, teachers’ fears pertaining to a perceived lack or loss of control is described (e.g. Carrillo & Flores 2020). Concerning the integration of social media, van den Beemt et al. (2020, p. 43) report additional barriers related to privacy, security, cyberbullying, and ethics. In conclusion, rather than offering a systematic approach towards technology integration, the reviews highlighted the need to take a closer look at the context of teaching and consider the interdependency of a variety of factors. A broad consensus exists that technology integration is promoted by external support via professional development measures as well as by supportive school environments.

#### Technology acceptance

Technology integration and application are closely linked with technology acceptance (Davis, 1986). In their meta-analysis, Scherer and Teo (2019) examine teachers’ technology acceptance in light of the theoretical implications of the TAM. Several other reviews also refer to and discuss individual or multiple assumptions of this framework to explain teachers’ intentions to integrate technology or their actual use of it. In relation to the model, researchers report that a number of factors directly influence technology integration, including perceived usefulness (PU; n = 3), perceived ease of use (PEOU; n = 2), and, most prominently, attitude towards technology (ATT; n = 8). In their meta-analysis, Scherer and Teo (2019) conclude that all relations within the TAM exhibit statistical significance, and they note the validity of PU, PEOU, and ATT in predicting technology integration.

Additionally, researchers have identified a variety of moderator variables that affect teachers’ acceptance and integration of technology. Scherer and Teo (2019) differentiate these variables as “organizational factors,” “technological factors,” and “individual factors” (p. 92). Among organizational factors, the studies highlight three contextual areas that affect teachers’ technology acceptance and integration: school type and culture, grade level, and subject area. These areas as well as their interdependency are reported to directly affect technology acceptance and, via this, technology integration (Spiteri & Chang Rundgren, 2020; Carrillo & Flores, 2020) focus on teaching and learning practices and highlight the need to differentiate various organizational situations, such as online teaching. Regarding technological factors, Scherer and Teo’s (2019) meta-analysis offers no statistical explanation of the effect of technology in general vs. specific technologies on the structural parameters of the TAM. Their meta-analysis, however, did not examine differences between specific technologies. Tondeur et al. (2012), meanwhile, discuss the advantages of specific technology education courses in transferring and implementing specific digital tools in future classrooms. Finally, teachers’ individual factors (i.e., gender, age, cultural background, intellectual capabilities, experience, subjective norms, and pedagogical beliefs) feature prominently in the results of several reviews. For example, Spiteri and Chang Rundgren (2020) report that technology acceptance/integration was influenced not by a teacher’s age but rather by teaching experience. In summary, while an abundance of variables on various levels is presented, previous reviews most often focused the influence of teachers’ personal attitudes towards technology in understanding technology acceptance in teaching.

### Future research

To answer our last research question, we examined the calls for future research in the individual reviews and identified the following five areas:

1. *Understanding context* To further develop the understanding of teaching and learning in diverse (digital) contexts, future research should go beyond the mere identification of contextual factors and critically examine how and why these factors (may) influence teachers’ digital literacy and/or willingness to integrate digital tools (Chai et al., 2013; Rokenes & Krumsvik, 2014; Rosenberg & Koehler, 2015; Scherer & Teo, 2019; Sherman et al., 2010; Starkey, 2020; Tondeur et al., 2017; van den Beemt et al., 2020; Voogt et al., 2013). Teachers’ pedagogical beliefs are highlighted, with the reviews repeatedly encouraging future research to take this personal factor into consideration (Carrillo & Flores, 2020; Pettersson, 2018; Tondeur et al., 2017).
2. *Process and outcome* Several reviews describe a lack of critical reflection in the included studies concerning the processes and specific outcomes of strategies and interventions related to teachers’ role in digitalization (Abbitt, 2011; Carrillo & Flores, 2020; Sherman et al., 2010; Tseng et al., 2020; van den Beemt et al., 2020). In this context, presenting and discussing best-practice strategies and focusing on practical learning areas, such as learning design, are suggested to benefit future research.
3. *Variety in methods* The reviews also demand (more) diversity in the methodological approaches to examining teachers’ digital literacy. More specifically, the results highlight the need for more case studies, interventional or experimental designs (Aydın & Gürol, 2019; Kay, 2006), research using mixed methods (Aydın & Gürol, 2019; Chai et al., 2013; Tondeur et al., 2017; van den Beemt et al., 2020; Wang et al., 2018; Willermark, 2018), and research employing longitudinal designs (Scherer & Teo, 2019; Tondeur et al., 2017; Wilson et al., 2020).
4. *Holistic perspective* Next in importance to teachers’ role in the process of digitalization, the reviews call for further research based on a more holistic examination of education. In this context, the reviews call for studies that consider the perspectives and effects of students (Aydın & Gürol, 2019; Chai et al., 2013) and school leadership (Fernández-Batanero et al., 2020; Pettersson, 2018).
5. *Clarifying concepts* Several authors also lament the lack of clear definitions and conceptualizations of specific terms or concepts (e.g., digital literacy, TK). This is discussed in conjunction with a call for improvement and agreement within the scientific community in future research (Kay, 2006; Voogt et al., 2013; Willermark, 2018).

## Discussion

In synthesizing the 23 selected reviews, we found an abundance of evidence highlighting the importance of research on teachers’ role in the process of digitalization. Our goal was to refine this knowledge base by combining these reviews “under one umbrella.” Instead of repeating searches, assessing the study eligibility of included articles, etc., we have distilled the findings of at least 1062 studies over the past 40 years that examine specific aspects of teachers and their role in the digitalization of education, offering an exclusive overview of past research on a meta-level, enabling a critical discussion thereof and proposing steps to pursue in upcoming years.

The holistic approach of our umbrella review examining digitalization of education from a teachers’ perspective offers the unique opportunity to discuss parallels and links between diverse theoretical approaches. As a result of this inclusive approach, we found that the requirements and strategies proposed for developing digital literacy and the integration of digital technologies into teaching appear to be strikingly similar (see Chaps. 4.2.3, 4.2.4, & 4.3.1). Although previous research has shown that digital literacy correlates positively with the integration of technology in teaching (McKnight et al., 2016; Starkey, 2020), we highlight that research so far put a sole focus on one of the two. Examining and better understanding the connection and dependences between these two areas could help clear up ambiguities.

We further found that the reviews highlight the necessity of discussing and reflecting on (existing) approaches and requirements for developing digital literacy as well as integrating technology into classes. The reviews identify and present an abundance of strategies for “developing” digital literacy and “supporting” technology integration (see Chaps. 4.2.3 & 4.3.1) but provide no evidence of the actual impact of these strategies. Based on the findings of our umbrella review, we recommend a critical discussion, application, and evaluation of these strategies in practice as a holistic approach involving the scientific community, schools, and policy representatives. In this sense, several reviews often lacked a clear theoretical background which could support their respective research focus.

This appears to be the case for the successful integration of technology in teaching. While an abundance of moderator variables for technology acceptance are mentioned in the reviews (Chap. 4.3.2), the reviews lack a discussion of the results and associated implications. In addition, as the findings show that multiple concepts are used to define digital literacy and that the TPACK model lacks clear definitions of the individual knowledge domains (Chaps. 4.2.1 & 4.2.2), we believe it is essential to more clearly define the concepts applied in the analysis of digitalization in education. The (further) development of the TPACK framework as proposed by Willermark (2018) represents a first step in this direction. In particular, a shift from TPACK as knowledge to TPACK as competence may offer the potential to better understand the appropriate applications in practical teaching.

Because education relies on a large, complex network of involved stakeholders (e.g., teachers, students, leadership, parents, policy makers), future research should consider multiple perspectives. For example, Tondeur et al. (2012) suggest the collaborative development of a technology plan for teacher education programs. Standardized self-evaluation tools, such as SELFIE,^2^ enable looking from multiple perspectives at schools’ status quo in examining the proficiency of students, teachers, and leadership in applying digital tools (European Commission, 2020).

### Implications

Our review’s findings have practical implications for schools, teacher education, and institutions offering professional development services to in-service teachers. In addition, we highlight implications for the research community in critically reflecting independent research on digital literacy and technology integration. First, the findings concerning schools highlight the pivotal role (and responsibility) of school leaders in translating the potential of digitalization into specific goals (Chap. 4.2.4). In line with McKnight et al. (2016), we encourage school leaders to proactively support teachers in further developing their digital literacy and integrating technology into classes. Rather than implementing general regulations and measures across school types or districts, our results underline the need for school leaders to consider the particular organizational, technological and individual factors of their school and staff (Chap. 4.3.2). This can be a starting point in taking a holistic approach to the creation of digitally competent schools, with leadership as key stakeholders in this complex system of education (Pettersson, 2018; Sailer et al., 2021).

A second key implication is that institutions of teacher education must act to adequately prepare preservice teachers for the 21st -century classroom. Responsible persons in teacher education programs need to embrace their status as role models, as our findings underline the importance of leading by example (Chap. 4.2.3). Integrating digital technologies into preservice teachers’ instruction both increases their digital literacy and prospectively motivates them to integrate technology into their future teaching.

Third, teacher professional development should be seen as an important resource for developing in-service teachers’ digital literacy (Chap. 4.2.4) as well as showcasing and teaching best practices for the integration of digital technology into classes (Chap. 4.3.1). According to the findings, both general formats for developing TK and subject-specific (TCK) and pedagogical formats (TPK) need to be addressed. Finally, we encourage dedicated sessions for school leaders to support them in the individual and complex process of digitalizing their schools.

Finally, we strengthen the need for the research community to critically reflect the current status of as well as the approach towards research on the teachers’ role in digitalizing education. While the reviews did a good job in synthesizing the abundance of specific studies, the current findings offer little practical support for schools, teacher education programs and institutions offering professional development measures. Rather than repeatedly examining the status of digital literacy or technology integration of a specific cohort of teachers, this review implies the critical role of the research community in actively supporting and shaping digital transformation processes. The identified areas for future research (Chap. 4.4) mark a starting point for the next phase of research.

### Limitations and recommendations for future research

Applying an umbrella review allowed us to synthesize the current state of research in an efficient and pragmatic manner. With this method, we can assess whether reviews aligning in topic independently reflect similar results and arrive at comparable conclusions (Aromataris et al., 2015). We acknowledge, however, that this approach also bears some risks. While the systematically selected reviews might align in topic, the reviews potentially examine a variety of different research questions, include different target groups, and differ in their timeline coverage and hence, might not be fully exhaustive (Happe et al., 2021). This could explain why 890 of the primary studies appear in only one of the selected reviews. Furthermore, as is the case for other variants of systematic reviews of research, the limitations of this umbrella review relate to subjective decisions of the authors concerning (a) the inclusion and exclusion of articles and (b) the inductive, thematic analysis of the included reviews. In the case of the former, the authors followed a strict, transparent protocol with appropriate quality appraisal to ensure the inclusion of all available reviews in the field (Aromataris et al., 2015). The thematic analysis, meanwhile, followed an iterative deductive and inductive coding process based on the existing literature, the specific research questions, and frequent discussions between the authors to ensure rigor (Braun & Clarke, 2006).

For future research, we highlight the need to adopt holistic perspectives and to consider context at all levels. We believe that research focused on the integration of specific types of technology (as proposed by Scherer & Teo 2019) or on the differences in types of participation (active vs. passive) (as suggested by Sailer et al., 2021) will increase our knowledge and understanding of the challenges and strategies related to integrating digital technology in education.

In addition, recommend that future studies draw upon and apply specific theoretical frameworks in their research. In our umbrella review, 11 reviews did not link their research to a specific theoretical framework (see Table 1). In line with Darling-Hammond (2006), we argue that theory must be applied to strengthen the field’s legitimacy to inform future policy development in education. In this light, we also recommend that researchers follow and report transparent research methods to (better) establish the applicability and transferability of results.

As our findings reveal a strong link between digital literacy and technology integration, we challenge future studies to further analyze this assumption by comparing and triangulating data of these two constructs. This could lead to further refining the usefulness of theory in understanding processes and the interaction of teachers’ digital literacy and technology integration.

## Conclusion

Teachers are central to the process of digitalizing education, so this umbrella review summarizes 40 years of research on their role in that process. The 1062 studies included in the 23 examined reviews make possible a sweeping overview of previous research as well as an outlook for future studies. We found broad variation in the conceptualization of digital literacy and described various approaches to successfully developing digital literacy and integrating digital technologies as well as parallels between these two distinct research areas. Finally, we examined and synthesized the calls for future research in five areas: understanding context, (critically) reflecting on processes and outcomes, variety in methodological approaches, diversity of perspectives, and clarifying concepts.

## Footnotes

1 We thank the authors who, upon request, sent us their complete lists of studies included in their analyses.

2 Self-reflection on Effective Learning by Fostering the use of Innovative Educational Technologies.
